# Process and impact of patient involvement in a systematic review of shared decision making in primary care consultations

**DOI:** 10.1111/hex.12458

**Published:** 2016-05-12

**Authors:** Catherine Hyde, Kate M. Dunn, Adele Higginbottom, Carolyn A. Chew‐Graham

**Affiliations:** ^1^ Research Institute for Primary Care & Health SciencesKeele UniversityKeeleUK; ^2^ West Midlands Collaboration for Leadership in Applied Health Research and Care (CLAHRC)UK

**Keywords:** analgesia, musculoskeletal pain, primary care, public involvement, shared decision making, systematic review

## Abstract

**Background:**

Patient and public involvement and engagement (PPIE) in systematic reviews remains uncommon, despite the policy imperative for patient involvement in research. The aim of this study was to investigate the process and impact of collaborating with members of a patient Research User Group (RUG) on a systematic review about shared decision making around prescribing analgesia in primary care consultations.

**Methods:**

Five members of an established patient RUG collaborated with researchers undertaking a systematic review with narrative synthesis, through workshops held at three time‐points. These addressed the following: designing the protocol, interpreting the results and planning dissemination. Support from a RUG coordinator and user support worker facilitated collaboration throughout the review process. Researchers reflected on how PPIE modified the review at each time‐point.

**Results:**

RUG members identified factors important in shared decision making around analgesic prescribing additional to those initially proposed by the research team. Search terms and specific outcomes of interest were amended to reflect these additional factors. Thirty of the 39 patient‐identified factors were absent in the published literature. The categories of factors identified were used as a framework for the narrative synthesis and for reporting results. RUG members prioritized options for disseminating the results.

**Conclusion:**

PPIE collaboration throughout the systematic review impacted on the scope of the review, highlighting gaps in the literature that were important to patients. Impact on interpretation and dissemination of findings ensured the review directly reflected patient priorities. Challenges and strategies to facilitate PPIE involvement in systematic reviews and suggestions for future researchers are highlighted.

## Background

The importance of patient and public involvement and engagement (PPIE) in health‐care research is recognized internationally.^1^, ^2^, ^3^, ^4^, ^5^ Patient and public involvement has been defined as ‘doing research ‘with’ or ‘by’ the public, rather than ‘to’, ‘about’ or ‘for’ the public’,^6^ and patient engagement as ‘where information and knowledge about research is provided and disseminated’.^7^ In addition to ethical and political arguments for the public having a voice in health‐care research, there is evidence that PPIE can impact on research questions, methods, dissemination of findings and engagement with local communities.^4^, ^8^, ^9^, ^10^, ^11^ Suggested positive effects of PPIE include prioritizing research topics, clarifying language in invitation letters, providing a wider perspective in data analysis and designing outcomes more relevant to patients.^8^, ^11^, ^12^ In the UK, PPIE is now integral to government‐funded health research, and a national advisory group, INVOLVE,^7^ provides guidance and examples of best practice for involving patients in health‐care research and for reporting patient involvement.^11^ Methods of implementing PPIE range from consultation, when researches seek the views of the public on key aspects of the research, to collaboration, an on‐going partnership throughout the research process, to publicly led research, in which lay people design and undertake the research.^9^ Current policy encourages PPIE using these different methods and within all research study designs.^7^, ^13^, ^14^

Systematic reviews ‘aim to identify, evaluate and summarize the findings of all relevant individual studies, thereby making the available evidence more accessible to decision makers' (Ref. 15, p5). Systematic reviews are the highest level of secondary research (which re‐examines the previously collected data^15^) investigating the effectiveness of health‐care interventions.^16^ As systematic reviews inform healthcare policy and guidance, PPIE in reviews is likely to have an important influence on healthcare delivery. Despite this, examples of patient involvement in systematic reviews remain rare and of varying detail and quality.^17^ Boote *et al*. describe a developing consensus of good practice for PPIE in health research 17, 18, 19 and best practice, and reporting guidelines have been developed.^20^, ^21^, ^22^ Our study aimed to integrate PPIE into a systematic review.

The review into which PPIE is integrated explores the factors affecting shared decision making around prescribing analgesia for musculoskeletal pain. Shared decision making (SDM) is the process whereby a health‐care professional and patient share information, and the patient is supported to consider management options.^23^ Musculoskeletal (MSK) pain includes both pain due to chronic conditions that have a specific diagnostic label such as osteoarthritis, and pain due to symptom labels such as ‘low back pain’ that may be chronic, episodic or acute in nature. MSK conditions remain one of the leading causes of disability worldwide,^24^ and half of those with an MSK condition feel that pain is the worst aspect of their condition.^25^ Many people with MSK pain take less than the therapeutic or prescribed dose of their analgesic,^26^, ^27^ and some have inadequate analgesia.^28^ Shared decision making within consultations may enable patients to be more active in prescribing decisions around their analgesic medications.^29^ This research aims to explore a collaborative approach to PPIE, in the context of a systematic review, by recognizing the particular expertise that patients have.^30^

### Objective

To describe the process and impact of involving patients in a systematic review and narrative synthesis of shared decision making around prescribing analgesia for musculoskeletal pain in primary care consultations.

## Method

To clarify the context of PPIE, the systematic review is briefly described. Methods of the systematic review are found in Box 1 and summary results in the results section in Box 2. The process of involving RUG members in the systematic review and how the changes made to the review were recorded are described below**.**

Box 1 **Details of methods of the systematic review of factors affecting shared decision making around prescribing analgesia for musculoskeletal pain in primary care consultations**

The search covered four broad topic areas, adapted from existing filters where possible for: primary care,^31^ musculoskeletal,^32^ shared decision making 33 and prescribing or analgesia.

Ten electronic databases were searched with follow‐up searches.

Key inclusion criteria were as follows: (i) primary care physicians and non‐pregnant adults (over 18 years old) with MSK pain; (ii) primary research studies focusing on consultations; (iii) sharing the decision of prescribing analgesia.

Outcomes of interest were factors that did or did not affect shared decision making around prescribing analgesia for musculoskeletal pain.

Quality of studies was assessed using existing criteria for quantitative and qualitative studies.^34^, ^35^ Studies were not excluded on the basis of quality, but methodological limitations informed the narrative synthesis. Results from full manuscripts were integrated into a narrative synthesis.^36^, ^37^

Box 2 **Results of the systematic review of factors affecting shared decision making around prescribing analgesia for musculoskeletal pain in primary care consultations**

Eight studies met the inclusion criteria (see Box 1 for details of inclusion criteria). Key messages suggested by the review were that (i) Patients' requests increase the likelihood of that medication being prescribed, and (ii) GPs attitude to the prescribing of opioid analgesics is controlling, due to legal and ethical concerns around prescribing opioids such as potential addiction.

Several other factors were investigated but the quality of the studies and strength of evidence was such that it was not possible to conclude whether those factors had an effect or not on SDM. For example, patient age and relationship with the GP may be important in SDM.

Thirty of the 39 factors identified by RUG members were absent in published studies included in the review (see Table S1, Additional Supplementary Information). Included studies did not investigate factors identified by RUG members in two categories of factors: ‘Emotion’ and ‘Condition’. Three factors from eight identified within the category ‘Impressions of the GP’ were found in the included studies: this was the most well investigated category.

### Systematic review method

The systematic review addressed the question ‘What factors affect shared decision making around prescribing analgesia for musculoskeletal pain in primary care consultations?’ Further details of the methods of the review are below in Box 1, and the full protocol is available from the lead author.

### Involving patients in the review process

Five members of an established patient research user group (RUG) collaborated with the researchers in the review process. RUG members of both genders, with a range of ages and musculoskeletal conditions, were recruited. Further information on the RUG and structure of support at the Research Institute has been reported in a case study.^38^

A RUG coordinator (RC) and a user support worker (USW) supported RUG members and liaised with the research team. The RUG support team were involved prior to preparation of the study protocol, giving advice on the suitability of the study for the RUG, agreeing aims and design, and providing written training material. The support team suggested having one of the support team and one researcher coordinate activities, as informed by their experience of different ways of working with researchers. The USW (Author AH) therefore acted as a point of contact for RUG members and facilitated meetings throughout the systematic review.

To inform the systematic review, the researchers and RUG support team planned workshops at three key points in the review process: when designing the protocol, interpreting the results and planning the dissemination. The workshops were planned by the researchers and reviewed by the RUG support team. Each three‐hour workshop included breaks and used a mixed format of presentation with discussion and small group techniques^39^ to ensure all RUG members could make a contribution. All authors agreed that having all three researchers in meetings with five RUG members would likely have been restrictive. Therefore, one researcher (Author CH) worked with the RUG members and support team throughout the systematic review process.

The first workshop explored RUG members' understanding of systematic reviews. RUG members were asked to discuss the research question and draw on their own experiences, share stories and describe key factors they thought were important to patients in sharing the decision of prescribing analgesia for musculoskeletal pain in primary care. At the second workshop, CH presented the preliminary results of the search and synthesis. RUG members were invited to critically discuss and plan how to share the results. At the final workshop, RUG members were asked to discuss and agree the final results of the systematic review and plans for further research and dissemination. After each workshop, notes were written up, checked by the USW and distributed to the RUG members with a comment sheet for them to return. RUG members were updated on the impact of their contribution via a newsletter and through discussion at each subsequent workshop. For all workshops, the RUG members were offered reimbursement for their time and travel as recommended as good practice for sustainable patient and public involvement in research.^40^, ^41^

### Recording the impact of patient involvement

To record the impact of PPIE, the researchers documented how the review changed at each stage using a before and after technique. Prior to the workshops, researchers discussed and documented their viewpoints and preliminary decisions. In the workshops, RUG members' perspectives were discussed, and similarities and differences between these and researcher perspectives were debated. Following each workshop, CH liaised with the research team (Authors CCG and KD) to discuss the results and implications of the discussions and how these could affect the review. RUG members were updated about changes to the review and given opportunities to respond.

## Results

Involving patients in the systematic review was feasible, and we successfully conducted three workshops with the RUG members. The aim, process and impact of the RUG members' involvement are summarized in Table 1. Impacts resulting from each workshop are described with specific details in the text below. Results of the systematic review itself are described in Box 2. Challenges and strategies for facilitating PPIE in systematic reviews are summarized in Table 2.

**Table 1:** Process and impact of the patient involvement in the systematic review of factors affecting shared decision making

Aim of patient involvement	Process of patient involvement	Impact of patient involvement
Preparation for review	Exploring relevance and importance of question with RUG coordinator and feasibility of involving members in research	Potential importance of review question to patients established
	Meeting with RUG coordinator and user support worker to establish exact aims and design of member involvement	Clear aims for and design of RUG involvement established, this facilitated application for funding to support review and patient involvement
Refining the scope of the review	Workshop 1 Discussing relevance of the research question to patients	Importance of review question to patients established
	Identifying factors important to patients in sharing decisions about prescribing analgesia	Additional factors of importance identified and review protocol & data extraction forms amended to reflect these
Interpreting the review preliminary findings	Workshop 2 Critiquing the results of review	Reassurance that RUG members raised ideas around relevance of literature that were similar to the researchers'
	Feedback on how well patients' views and priorities had been integrated	Reassurance that patient priorities had been reflected in review process. Categories identified by RUG members were used as a framework for the narrative synthesis
Interpreting the review findings	Workshop 3 Agreeing of final results of review	Reassurance that patient priorities had been reflected in review process. RUG members highlighted additional factors that were poorly represented in literature
Disseminating findings and engagement	Workshop 2&3 Planning how to share results	Results were targeted at practitioners, as RUG members felt this was most important
	Agreeing dissemination of the results & discussing impact of group's involvement	RUG members and support team participated in dissemination of the review findings
	Discussing how factors important to patients may be observed in consultations	Next stage of the research informed by patient perspective and priorities

**Table 2:** Challenges of involving PPIE in systematic reviews, strategies for facilitating involvement and suggestions for researchers

Challenges as identified by Boote *et al*.^17^	Strategies the researchers used to manage challenges	Researchers' suggestions for managing challenges
Time pressures (developing PPIE network, building trust, allowing for involvement at more than one time‐point)	Used established PPIE network	Use an established PPIE network if this is possible. It may be easier to recruit people with a different condition using an existing support network
	Contact was made with PPIE coordinator 18 months prior to review start date	Start PPIE as early as possible – that is at question formation stage
	PPIE took place over 10 months	Recognize that PPIE may extend the research timeline
Resources (funding and time)	Funding application for PPIE	Apply for funding for PPIE ***and*** engagement activities, for example members attending conferences
	Advice from PPIE in funding application	Seek advice for funding application (INVOLVE, Research Design Service http://www.rds.nihr.ac.uk/public-involvement/)
	Reimbursement available at INVOLVE rates for those who wished it	Offer reimbursement for time and travel at recommended rates
	Allowed time for PPIE (100 h) and admin support (25 h)	Resource time for PPIE and admin support
	Researcher time allowed for (50 h)	Allow for researcher time for developing materials, writing up notes, discussing impact of PPIE
Continuity	Having a lead PPIE and researcher	Have both a lead researcher and if possible PPIE coordinator
	Recognized members are more likely to have health issues and may wish to limit involvement or be involved at different stages	Be flexible in how and when members are involved in on‐going projects. Make expectations and flexibility clear at the beginning
	Rearranged a meeting that fewer than four members could attend	Decide on a minimum number of members needed, recruit more than this and allow time for possible rearrangement of meetings
	Encouraged members to continue involvement, agreed realistic outcomes initially and gave written and verbal updates of progress	Encourage continued participation, agree realistic outcomes initially and give updates on progress
Concerns about group dynamics (power balance between members and researchers or within the group)	Selecting a diverse range of members who already had worked together	Consider using members who have already successfully worked together
	Having a lead PPIE and researcher working with each other throughout the project and colocated	Recognize power relationships can be an issue to manage. Clearly recognize and appreciate PPIE members expertise
	Used small group techniques to create a relaxed atmosphere & encourage individual contributions	Consider researcher training in small group techniques and debrief with any facilitators after meetings
	Allowed time before, during and after meetings for members and researchers to discuss on a social level and raise any concerns	Allow time before, during and after meetings for members and researchers to discuss on a social level and raise any concerns
	Encouraged members to return written, anonymous comment sheets to raise additional information, and any concerns	Give members different ways of expressing their opinion and any concerns (written, online, within‐group, individually)
Research Ethics Committee involvement	Sought national and expert guidance that PPIE does not require ethics approval	Cite national^42^ and expert guidance that PPIE in systematic reviews does not require ethics approval
‘Representativeness’ of members involved	Coordinator &USW with expertise including engagement, supported RUG members	Consider a model of PPIE with specific support, or recruiting members in different ways so members feel comfortable in their role
	Establishing that having members with prior research experience would be useful as the review was more complex	Consider the research and condition experience needed for different aspects of the project, dissemination and engagement
	Training and glossary developed locally, and discussion with members about their learning needs in workshops	Discuss members' training needs, recognizing individuals will have different experiences. Consider sharing existing training resources

### Workshop 1: Refining the scope of the review

The RUG members influenced the scope of the review by identifying factors that may affect SDM around prescribing analgesia for MSK pain, additional to those identified by the researchers. The researchers had highlighted factors potentially affecting SDM through preliminary literature searching and their clinical experience (CH and CCG). The researchers categorized these into ‘GP’ factors, ‘Consultation’ factors and ‘Patient’ factors. An example of a factor from the ‘GP’ category is that a GP's past experience of using a specific analgesic can influence SDM around prescribing that analgesic.

RUG members identified factors important to patients in sharing decisions about prescribing analgesia. They developed categories which were similar to the researchers, with additional categories of ‘Medication’ and ‘Emotion’. Rather than a category of ‘GP’ factors, patients discussed factors both of their ‘Impression of the GP’, such as the GP's ability to listen and explain, and their ‘Impression of external influences that affect the GP’, such as guidelines. The full list of 39 factors in seven categories identified by RUG members is available as supplementary information. The additional factors identified by the RUG members resulted in two changes to the review search strategy. The first change was that search terms for SDM were amended to reflect additional factors such as ‘Emotion’. The second change was that the researchers specifically looked for additional factors identified by RUG members in the full‐text studies included in the review.

### Workshop 2: Interpreting the review findings

RUG members critiqued the provisional results of the review and identified limitations of the review literature. An example of RUG members' interpretation of the results is that RUG members were surprised at the poor representation of factors that they had identified. RUG members and researchers both identified a lack of SDM research in patient populations as a limitation in the existing literature. RUG members identified an additional limitation in the review literature that patients involved in studies may have felt uncomfortable raising concerns about their medical care, particularly if the researcher also had a clinical role.

The categories of factors identified by RUG members were used as a framework for the narrative synthesis and for reporting results. The patient perspective was integrated into the implications of the review by translating concerns RUG member raised into suggestions for further practice. For example, RUG members' surprise at the lack of studies of SDM with patient populations emphasized gaps in the literature for further study.

### Workshop 3: dissemination of findings and reflections on engagement

Initial discussion of dissemination strategies focused on reaching patient audiences to empower patients when consulting a GP, to suggest medications that the patient had heard about or thought might be useful. RUG members discussed the finding that a patient suggesting medication to a GP increased the chance of that medication being prescribed, which may empower some patients. However, the RUG members raised concerns that patients suggesting medication to a GP may lead to conflict within the consultation. Therefore rather than aiming to disseminate findings with patients, we prioritized dissemination and engagement with GP audiences.

RUG members and the support team planned their own roles in engagement including reviewing abstracts, presentations and publications, attending and giving presentations and contributing the patient's perspective to discussions at conferences. Gaps in the literature identified in the review and factors identified by the RUG members informed a subsequent phase of research: a secondary analysis of digitally recorded GP consultations exploring how decisions are shared in practice. PPIE continued into this phase of the research.

### Challenges of, and strategies facilitating, PPIE in systematic reviews

A number of challenges of involving PPIE in systematic reviews such as time pressures and managing power dynamics have been identified in a review of case examples.^17^ Strategies used by the researchers to manage these commonly identified challenges and suggestions from their experience of integrating current recommendations for good practice around PPIE in a systematic review are described in Table 2. Key strategies for addressing challenges included continuity with a PPIE lead in the PPIE support team and researcher team, and applying for specific funding to support PPIE time and resource requirements.

## Discussion

This paper reports PPIE collaboration at three time‐points in a systematic review and reflects on the impact on the review. Altering the search strategy to reflect additional factors identified by PPIE members highlighted gaps in the published literature around issues important to patients which would have otherwise gone unreported. PPIE throughout the review ensured that the patient's perspective was integrated into the protocol design, interpretation of the results and planning and prioritizing dissemination.

This study applied previous research on good practices for PPIE in health‐care research^18^, ^19^, ^20^, ^22^ and in systematic reviews.^17^, ^21^ We used case examples described by Boote *et al*.^17^ to anticipate and report common challenges in PPIE. We found that the work by Boote *et al*.^17^ provided a comprehensive framework for the challenges we faced. Placing the patients' perspective alongside the scientific paradigm can increase ‘credibility’ with a multilayered understanding in research.^43^ Concerns that PPIE could be a source of research bias have been described.^9^ This study demonstrates how the patients' perspectives both agreed with and differed from the researchers' perspectives. Two approaches towards PPIE in this study address concerns of bias. Firstly, clear description of the review before and after each time‐point at which PPIE occurred so that the influence of PPIE was recorded and reported clearly. A second approach is linking aims of PPIE with the process and impact of PPIE so that the intention, application and influence of PPIE in the research is apparent. These transparent approaches to PPIE may prove useful for further enquiry into the impact of PPIE on research.

PPIE has been performed in several other systematic reviews in a health‐care context, but details of impact of PPIE on systematic reviews are described in separate reports^8^, ^44^, ^45^ rather than in the reviews themselves.^46^, ^47^, ^48^ It has been suggested that the structure of reviews change so that the process and impact of PPIE are routinely reported.^8^ This paper provides an example for reporting PPIE within a systematic review, and the PIRICOM report is a further illustration.^8^

Previous research has reported PPIE in dissemination through patient‐focused publications and the popular media in addition to traditional conferences.^44^, ^48^ Our study is to our knowledge the first to report the PPIE in prioritizing dissemination and engagement activities in a systematic review within health care. Involving patients in prioritizing the dissemination and engagement plan resulted in early identification of challenges in implementing findings from the review with patient audiences.

### Strengths and limitations

Strengths and limitations of the study are focused around two issues, the ‘representativeness’ of PPIE and the extent of PPIE integration within the review. How well those involved in PPIE ‘represent’ the wider community is a recognized tension.^9^, ^49^ Lay experience is ‘located within personal history and narrative logic’ and contains a ‘diversity of world views’[43 p513]. In order to capture this diversity, both who is involved and how they are involved influences the representativeness. Strengths of the PPIE representativeness in this study are that RUG members with different research and condition experiences were involved, supported by a team and working with one researcher. These RUG members could therefore potentially voice a wider range of experiences than individuals who are very well integrated into the research community. Secondly, the researchers used strategies throughout to facilitate sharing the experiences and narratives from all RUG members. Limitations in representativeness are that the RUG members had similar white British ethnicity and were all over the age of 45 years. Different groups such as carers or stakeholder organizations^10^ were not involved and including individuals from different populations and roles may have yielded differing factors and priorities. However, the factors RUG members identified were similar in number and scope to those identified in a systematic review of patient reported barriers and facilitators to SDM in health‐care consultations.^50^ This suggests that the RUG members identified factors that were broadly relevant to patients.

The extent of PPIE integration into the review process also raises strengths and limitations of the study. It is a strength of the study that PPIE occurred at the early stage of question development, and continued throughout the review process. Having one of the PPIE support team and one researcher acting as points of contact is a strength as it facilitated clear communication and clarity of roles. Tokenistic PPIE in health‐care research is a concern,^12^ and in this study, we aimed to truly value PPIE and took practical steps to manage challenges of PPIE and facilitate involvement throughout (see Table 2). However, lack of understanding of how PPIE members themselves felt about the extent of their integration into the study is a limitation. Although members did not drop out between workshops, which has been discussed as proxy for PPIE satisfaction,^51^ there was no evaluation of the experience of the PPIE members themselves and this highlights a gap in methods and tools for understanding PPIE member experiences.

### Recommendations

Future research exploring PPIE should use best practice guidelines and our recommendations when planning, implementing, evaluating and reporting PPIE in systematic reviews. This approach will enable researchers to build on what is already known in this area, anticipate and manage expected challenges, and document the impact of PPIE clearly. It will also facilitate comparison and synthesis of PPIE across reviews. The impact of PPIE reported in this study is on the systematic review itself. Further work remains to be done on evaluating the impact of involvement in reviews on PPIE members themselves.

## Conclusions

PPIE collaboration refined the scope of this systematic review and influenced interpretation and dissemination of the findings. Gaps in published literature important to patients were highlighted and the systematic review and its dissemination more clearly reflected patient priorities. PPIE in this systematic review built on existing good practice guidance and examples in design, implementation and reporting. Future researchers may draw on strategies discussed to integrate good practice into their work, including transparent description of PPIE impact and describing how common PPIE challenges are managed. As researchers respond to policy imperatives to integrate PPIE into their research, PPIE in systematic reviews should utilize best practice as described so that patient perspectives and priorities are clearly represented.

## Supporting information


**Table S1.** Table of factors identifed by RUG members and systematic review which affect shared decision making around prescribing analgesia for musculoskeletal pain in primary care consultations.
