# Supplementary material for: Process and impact of patient involvement in a systematic review of shared decision making in primary care consultations
Source: Health Expect. 2016 May 12;20(2):298–308. doi: 10.1111/hex.12458 (PMC5354055; doi:10.1111/hex.12458)
Supplement: Supplementary file 1 — Table S1. Table of factors identifed by RUG members and systematic review which affect shared decision making around prescribing analgesia for musculoskeletal pain in primary care consultations. [file HEX-20-298-s001.docx]

| **Supplementary information: Table of factors identifed by RUG members and systematic review which affect shared decision-making around prescribing analgesia for musculoskeletal pain in primary care consultations** | | | | | | |  |
| --- | --- | --- | --- | --- | --- | --- | --- |
| **Patient** | **Condition** | **Emotion** | **Consultation** | **Medication** | **Impressions of the GP** | **Patients perception of external influences that affect the GP** | |
| Mental state- e.g. depression | Diagnosis | Desperation of pain | **Time** | **Type of analgesia e.g. opioid** | **Taking time to listen & understand** | GP needing to follow guidelines | |
| Other medical problems | Severity of pain | Distress caused by pain | Emotional state at  beginning of  consultation | How it is used - e.g. patch, gel | Taking time to explain | How GPs prioritise problems | |
| Feeling like can't prioritise pain | Where the pain is | Fear of pain coming back & potential different causes | Other issues or problems to be discussed | Side effects potential & caused | Taking the problem seriously | **GP feeling they don't have enough time** | |
| Different wants and needs for information  Employment | One or more sites affected | Frustration in not being able to manage pain or know diagnosis |  | Other prescribed medication | How GP manages emotions, how the start the consultation | Lack of ability to do anything e.g. only give analgesia | |
| Fear of analgesia - side effects, addiction, masking the problem  Reason for coming to GP | Where condition is in 'diagnostic timeline' e.g. how long have had it, is there a diagnosis | ***Patient***  ***expressed emotion: anger, dejection, confidence*** |  | Safe potential analgesia  **Cost**  ***Medico-legal issues*** | **Continuity: relationship with GP**  **Attitude towards patients/ sharing decisions with patients**  Gender and age (for some members) | **GPs’ experience with different analgesia**  ***Prescribing habit***  ***Societal effect*** | |
| Past experience of pain & analgesia  **Assertiveness - in asking for analgesia** |  |  |  |  | ***Whether GP knows history***  ***Confidence and ability to communicate risk*** |  | |
| ***Preference for SDM*** |  |  |  |  |  |  | |
| **Legend *Italics*:** additional factors from the studies included in the review  **Underlined**: factors identified both by PPIE and studies included in the review | | | | | | | |
